# Assessing the resilience of stochastic dynamic systems under partial observability

**DOI:** 10.1371/journal.pone.0202337

**Published:** 2018-08-23

**Authors:** Jacopo Panerati, Nicolas Schwind, Stefan Zeltner, Katsumi Inoue, Giovanni Beltrame

**Affiliations:** 1 Département de Génie Informatique et Génie Logiciel, Polytechnique Montréal, Montréal, QC, Canada; 2 National Institute of Advanced Industrial Science and Technology, Tokyo, Japan; 3 Bloomberg L.P., New York, NY, United States of America; 4 National Institute of Informatics, Tokyo, Japan; 5 School of Computing, Tokyo Institute of Technology, Tokyo, Japan; Kungliga Tekniska Hogskolan, SWEDEN

## Abstract

Resilience is a property of major interest for the design and analysis of generic complex systems. A system is resilient if it can adjust in response to disruptive shocks, and still provide the services it was designed for, without interruptions. In this work, we adapt a formal definition of resilience for constraint-based systems to a probabilistic framework derived from hidden Markov models. This allows us to more realistically model the stochastic evolution and partial observability of many complex real-world environments. Within this framework, we propose an efficient and exact algorithm for the inference queries required to construct generic property checking. We show that the time complexity of this algorithm is on par with other state-of-the-art inference queries for similar frameworks (that is, linear with respect to the time horizon). We also provide considerations on the specific complexity of the probabilistic checking of resilience and its connected properties, with particular focus on resistance. To demonstrate the flexibility of our approach and to evaluate its performance, we examine it in four qualitative and quantitative example scenarios: (1) disaster management and damage assessment; (2) macroeconomics; (3) self-aware, reconfigurable computing for aerospace applications; and (4) connectivity maintenance in robotic swarms.

## Introduction

Originally coined in the context of environmental sciences and ecological systems, resilience has become a property of great interest for the study of complex systems. Although resilience is not easily defined, researchers agree that it is a fundamental characteristic of those ecosystems that are able to absorb extreme spikes and survive, albeit transformed. The insect populations of North-eastern American forests [1] are well-known examples of such resilient systems.

The focus of the artificial intelligence community has been, so far, on narrowing down the concept of resilience and formalizing it, for example in constraint-based and non-deterministic dynamic systems [2]. These approaches are extremely general and able to describe a plethora of real-world systems, but they have very limited predictive power. The transition models in non-deterministic dynamic systems resemble those of Markov chains and decision processes but, because they do not have probability distributions associated to transitions, they do not tell whether a future world is more likely than the others.

Succeeding in the definition and implementation of resilience has the potential to enable the creation of “resilient by design” systems. In computing engineering, for example, networks and robotics systems provided with resilient properties will possess the ability to absorb shocks and to transform in response to external attacks, while still providing their services.

By choosing to study resilience in the context of hidden Markov models, we extend the existing artificial intelligence research to take into account the unpredictability of the real world. This is essential to make our model consistent with the idea of a “random world” proposed by Holling [1]. In fact, conditional probability distributions can be seen as the stochastic extension of non-deterministic transition functions.

The choice of hidden Markov models is, hence, manifold. They allows us to (i) capture the unpredictability of the world’s evolution; (ii) account for the uncertainties in our perception of the world; and (iii) reason about the passing of time. All this, while keeping the complexities of the model and inference reasonably low. Despite their simplicity, hidden Markov models have demonstrated to be capable tools for daunting applications. Notable examples being genome phasing [3] and speech recognition—a task for which more complex approaches based on deep learning started matching their performance only in recent years [4].

In this work, we also expand the previous discussion about resilience with the element of partial observability, adding one more layer of complexity. In the end, the goal of our work is to provide the formal and algorithmic tools to efficiently answer queries such as: “what is the likelihood of requiring extra personnel in an emergency area over the next three days?”, “what is the probability that a worker robot will soon become disconnected from its assigned cluster?” or “with 99% confidence, what is the minimal number of neighbor links to maintain connectivity in an extremely noisy network?”.

## Related work

In a seminal paper from 1973, Holling introduced the concept of “resilience of ecological systems” [1]. In it, he draws a clear separation line between resilience and the more commonly used notion of stability. Resilient systems are not those systems that simply react to imbalances by quickly returning to equilibria. Instead, when perturbed, they are able to find new sustainable configurations. It is worth noting that Holling defines resilience in the context of what he calls “the random world”: an environment that is intrinsically stochastic. Developing these ideas, Walker *et al.* [5] define resilience as “the capacity of a system to absorb disturbance and reorganize while undergoing change so as to still retain essentially the same function, structure, identity, and feedbacks”.

Computer science has often looked at biology as a source of inspiration for the development of search algorithms, coordination mechanisms, and complex frameworks. The first attempt to develop a formal definition of resilience exploiting the tools of artificial intelligence was provided by Överen, Willsky, and Antsaklis [6] and, successively, further developed by Baral *et al.* [7] and Schwind *et al.* [2]. Our research is based on the formal description of the System Resilience- (SR-)model introduced by Schwind *et al.* [2]. When compared to the existing research [2, 7], the main distinctive trait of our work is in its integration of the ideas of probability theory. Our analysis of resilience is based on the probabilistic framework of hidden Markov models (HMMs). HMMs are often employed in applications such as signal and natural language processing. Nonetheless, they have also proven to be fruitful descriptive tools for many other complex dynamic systems [8].

Our methodology is closely connected to the sub-field of artificial intelligence that deals with probabilistic graphical models, dynamic Bayesian networks in particular. The two most common types of inference tasks for probabilistic graphical models—the larger family of frameworks HMMs belong to— are marginal and maximum a posteriori estimation (i.e. the computation of the distribution of a single variable and the most likely assignment of all variables, respectively). For these, efficient algorithms with convenient linear time-complexity have been identified [9]. In the following, we show that these queries are not sufficient to perform the kind of property checking demanded by our formal definition of resilience. An *ad hoc*, efficient algorithm to answer the necessary queries is detailed in S1 Appendix.

Hidden Markov models research has been prolific in multiple application domains. As we mentioned, HMMs have been used with success for genome phasing [3] and speech recognition. Tackling the problem of modelling the duration distributions of phonemes, Johnson noted that “a simple adjustment to HMM topologies is perhaps a more efficient solution [..] than more complex approaches” [10]. HMMs have also proven to been effective and, equally importantly, low complexity instruments for face recognition [11]. Because of their compact representation, HMMs can be quickly and efficiently compared one another with the aid of similarity measures [12]. Recent research has also exploited HMMs representation for the resilient filtering of Markov jump systems [13, 14].

With regard to applications, resilience has been, in recent years, a topic of interest for researchers in many different areas. Beyond ecology, these areas include economics, networking, critical and real-time systems, and swarm robotics—a domain that lies at the prolific intersection of computer engineering and biology. Researchers have been developing ways to formalize the robustness and resilience [15, 16] of networks of robots with respect to their most common tasks, e.g. consensus, flocking, and formation. Our work shares some terminology with this research and can also be used to address fundamental problems of swarm robotics (e.g. the one of connectivity). However, it is worth noting that the formal definition of resilience given here is not a domain-specific one and it could be used orthogonally with that, for example, of Saldaña *et al.* [16] (see the Application Scenarios section).

## Resilience and resilient properties in probabilistic models

This work re-interprets a formal definition of resilience (for dynamic systems) [2] using the probabilistic framework of hidden Markov models and enriching it with a cost function. In this section, we recall and combine together a number of definitions that are derived from recent research work on formal resilience in dynamic non-deterministic constraint-based models [2, 17] and timed probabilistic models [18].

The SR-model is a theoretical framework proposed by Schwind *et al.* [2] that combines elements of constraint-based systems and non-deterministic dynamic systems. It gives us a formal definition of resilience, as the unifying property arising from three simpler properties: 1) resistance, 2) functionality, and 3) recoverability. The SR-model consists of two separate formal descriptions for the kinematics and the dynamics of a system: the first is represented as sequences of pairs called “state trajectories” or SSTs:

$$\begin{array}{c} SST=\left(CBS_{0},\varsigma_{0}\right),\left(CBS_{1},\varsigma_{1}\right),\ldots ,\left(CBS_{i},\varsigma_{i}\right),\ldots \end{array}$$ (1)

The subscript index skims through the time steps. The symbol *CBS*_*i*_ represents a constraint-based system composed of a set of variables **X**_**i**_ and a cost function *κ*_*i*_:

$$\begin{array}{c} CBS_{i}=\left\langle X_{i}=\left\{X_{i}^{0},X_{i}^{1},\ldots ,X_{i}^{j},\ldots \right\},\kappa_{i}:D\left(X_{i}\right)\to R^{+}\right\rangle \end{array}$$ (2)

The second element in each pair, $\varsigma_{i}\in D\left(X_{i}\right)$, represents a complete assignment of the variables in **X**_**i**_: $\varsigma_{i}\in R^{|X_{i}|}$. Each SST corresponds unambiguously to a sequence of costs obtained by plugging-in each *ς*_*i*_ into its corresponding cost function *κ*_*i*_: *κ*_0_(*ς*_0_), *κ*_1_(*ς*_1_), …. The environment dynamics are described using non-deterministic Dynamic Systems (DSs):

$$\begin{array}{c} DS=\langle \text{CBS},A,m:\text{CBS}\times A\to P(\text{CBS})\rangle \end{array}$$ (3)

where **CBS** represents the set of all possible constraint-based systems *CBS*_*i*_, **A** is the set of actions available at each time step, and *m* is a non-deterministic transition function that, given the current CBS and an action, returns the set of possible constraint-based systems for the next time step.

The kinematic description of the SR-model (SSTs and sequences of costs) is central to the formalization of resilience and it is preserved in our proposed methodology. However, we prefer to discard the non-deterministic description of the dynamics in favor of a probabilistic approach based on hidden Markov models. Hidden Markov models (HMMs) can be seen as specific subset of both dynamic Bayesian networks (DBNs) and state-observation models [9]. HMMs have a single discrete state variable *S* and a single discrete observation variable *O*. A HMM is fully specified by the probability distribution of *S* at time −1, *P*(*S*_−1_), the conditional distribution of *O* given *S* at the same time step, *P*(*O*_*t*_ ∣ *S*_*t*_), and the conditional distribution of *S* given *S* at the previous time step, *P*(*S*_*t*+1_ ∣ *S*_*t*_) [19].

$$\begin{array}{c} HMM=\langle P\left(S_{-1}\right),P\left(O_{t}|S_{t}\right),P\left(S_{t+1}|S_{t}\right)\rangle \end{array}$$ (4)

HMMs are commonly used for the tasks of signal processing and speech recognition [19] because efficient (i.e. with computational time complexity that is linear with respect to the time horizon of the model) algorithms exist for: 1) the estimation of the probability distribution of *S*, also called the “hidden” variable, taking only assignments of *O* as input (filtering and smoothing algorithms); and 2) the identification of the most likely sequence of assignments of *S*.

To formalize resilient properties in the probabilistic context of a “random world”, HMMs offer the probabilistic reasoning of DBNs and the independence assumptions of state-observation models. We chose HMMs above other frameworks such as Markov decision processes (MDPs) and partially-observable Markov decision processes (POMDPs) because these lacked an explicit management of time and their decision layer was deemed unnecessary for the assessment of resilience.

The creation of a new framework to describe the resilience of stochastic, partially observable systems, requires, however, certain additional steps. First, we re-define the domain of the random variables *S* and *O* as the union of the domains of the set of variables of the constraint-based systems in **CBS**:

$$\begin{array}{c} \Omega \left(O\right)\subseteq \Omega \left(S\right)=\cup_{i}\;D\left(X_{i}\right) \end{array}$$ (5)

Without loss of generality, we impose a static cost function: $\forall i,\;\kappa_{i}=c:\Omega (S)\to {\mathbb{R}}^{+}$ and we introduce a sensor model that describes the imperfect observations of the set of variables: $P(O_{t}|S_{t}):\cup_{i}\;D(X_{i})\times \cup_{i}\;D(X_{i})\to [0,1]$. Because we are not interested in formulating a decision making problem, we drop the set of actions **A** from *DS* and we replace *m* with the conditional probability distribution that describes the probability of a set of variables evolving into another:

$$\begin{array}{c} P\left(S_{t+1}|S_{t}\right):\cup_{i}\;D\left(X_{i}\right)\times \cup_{i}\;D\left(X_{i}\right)\to \left[0,1\right] \end{array}$$ (6)

Putting these elements together with an initial probability distribution $P\left(S_{0}\right):\cup_{i}\;D\left(X_{i}\right)\to \left[0,1\right]$, our overall framework can be re-written as:

$$\begin{array}{c} c\text{-}HMM=\langle P\left(S_{-1}\right),P\left(O_{t}|S_{t}\right),P\left(S_{t+1}|S_{t}\right),c:\Omega \left(S\right)\to R^{+}\rangle \end{array}$$ (7)

**Fig 1:**
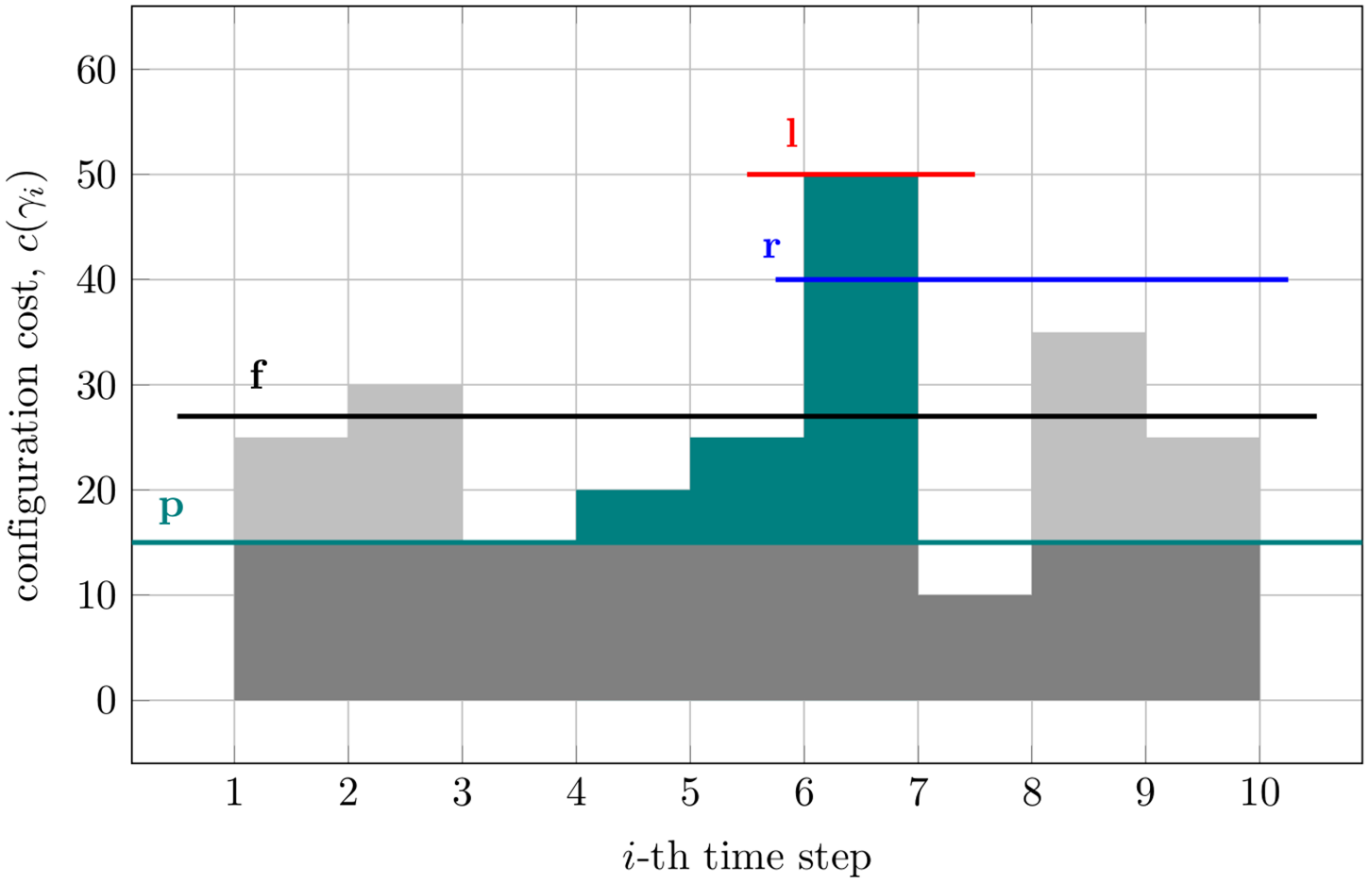
Cost trajectories and their properties. The example of a cost trajectory that is 50-resistant, 27-functional, 〈15, 50〉-recoverable, and 〈4, 40〉-resilient, according to the Definitions 1 to 4 and the Eqs 8 to 10 provided in this work.

In the SR-model, resilience is a boolean property of a state trajectories SST. It can be seen as a unifying property, combining different desirable behaviors of a dynamic system and arising from three simpler properties of state trajectories: resistance, functionality, and recoverability (see Fig 1).

### *l*-resistance

The resistance property expresses the fact that a trajectory never incurs in a cost that is larger than a fixed threshold. Therefore, this property is parameterized by this maximum acceptable cost.

**Definition 1**. *Given a state trajectory SST* = (*CBS*_0_, *ς*_0_), (*CBS*_1_, *ς*_1_), … *and a positive threshold* $l\in R^{+}$, *SST is said to be l-resistant if and only if each cost in its corresponding cost sequence is less than or equal to the threshold l*:

$$\begin{array}{c} \kappa_{i}\left(\varsigma_{i}\right)\leq l\;\;\;\;\forall \kappa_{i}\left(\varsigma_{i}\right)\in \left(\kappa_{0}\left(\varsigma_{0}\right),\kappa_{1}\left(\varsigma_{1}\right),\ldots ,\kappa_{n}\left(\varsigma_{n}\right),\ldots \right) \end{array}$$ (8)

This property must be satisfied whenever we deal with periodic, fixed budgets.

### *f*-functionality

The functionality property tells us if the costs of a trajectory are, on average, equal to or below a certain threshold. As in the case of resistance, this threshold parameterizes the property.

**Definition 2**. *Given a state trajectory SST* = (*CBS*_0_, *ς*_0_), (*CBS*_1_, *ς*_1_), … *and a positive threshold* $f\in R^{+}$, *SST is said to be f-functional if and only if the arithmetic average of the costs in its corresponding cost sequence is less than or equal to the threshold f*:

$$\begin{array}{c} {\left|SST\right|}^{-1}\cdot \sum_{i=0}^{\left|SST\right|}\kappa_{i}\left(\varsigma_{i}\right)\leq f \end{array}$$ (9)

This property is important when the operations we plan and our budget have different time granularity.

### 〈*p*, *q*〉-recoverability

The recoverability property concerns those systems in which costs over a certain threshold can be accepted, but only as long as the system is able to return within normal conditions before consuming a fixed, restorable, reserve.

**Definition 3**. *Given a state trajectory SST* = (*CBS*_0_, *ς*_0_), (*CBS*_1_, *ς*_1_), …., *and a positive threshold* $p\in R^{+}$ *and a positive budget* $q\in R^{+}$, *SST is said to be* 〈*p*, *q*〉-*recoverable if and only if every time the sequence of costs exceeds the threshold, it also returns below (or at) it before the cumulative offset surpasses the reserve*:

$$\begin{array}{c} \forall k\;s.t.\;\kappa_{k}\left(\varsigma_{k}\right)>p,\exists j>k\;s.t.\;\kappa_{j}\left(\varsigma_{j}\right)\leq p\wedge \sum_{i=k}^{j-1}\left(\kappa_{i}\left(\varsigma_{i}\right)-p\right)\leq q \end{array}$$ (10)

Systems with storage abilities—and that can use resources faster than they replenish them—are affected by this property.

### 〈*z*, *r*〉-resilience

Having explained the concepts of resistance, functionality, and recoverability, we can finally define the resilience of SSTs as a property-aggregating property.

**Definition 4**. *Given a state trajectory SST* = (*CBS*_0_, *ς*_0_), (*CBS*_1_, *ς*_1_), …., *a natural number* $z\in N^{*}$, *and a positive threshold* $r\in R^{+}$, *SST is said to be* 〈*z*, *r*〉-*resilient if and only if all its sub-trajectories of length z are r-functional*.

As it was observed by Schwind *et al.* [17], using this definition, resilience is strongly interconnected with the three previous properties: by setting the parameter *z* to 1 or |*SST*|, resilience becomes equivalent to *r*-resistance or *r*-functionality, respectively. Moreover, Schwind *et al.* [17] proved that “a finite SST is 〈*p*, *q*〉-recoverable if it is 〈*z*, (*p* + *q*/*z*)〉-resilient ∀*z* ∈ {1, …, |*SST*|}”.

## Complexity of efficient exact inference

In S1 Appendix, we describe how to use the c-HMM framework to define the random variables associated to trajectories of states, observations, and costs, i.e. the probabilistic analogues of SSTs. We then show that the probability of a trajectory of costs can be derived from those of trajectories of states. However, a major assumption of our work is that only trajectories of observations are available to study the partially observable stochastic system. Because of this reason, we introduce an efficient algorithm to perform the exact inference needed to find the probability of a trajectory of states from a trajectory of observations. The algorithm is also detailed in S1 Appendix. The most commonly used algorithms for exact inference in HMMs—the forward algorithm, the forward-backward algorithm and the Viterbi algorithm [9]—are all characterized by linear computational complexity. They target marginal and maximum *a posteriori* inference [19] and they are inherently suited for the processing of long chains of input [3, 10]. Indeed, for any new inference query we intend to introduce, we must aspire to compute it with linear time-complexity with respect to its input.

Here, we study our algorithm’s complexity starting from the consideration that its last step requires the multiplication of three separate probability values (factors) that we called ϒ_0_, ϒ_1_, ϒ_2_.

We assume that querying the sensor and the transition models for any of their elements involves a constant and negligible delay. The computation of ϒ_0_ is the quickest: starting with an initialization value of 1, we need to multiply it *T* times (the length of the time horizon of the assessment or prediction of a property) by the correct entry of the sensor model. Therefore, the time complexity of ϒ_0_ is *O*(*T*). The factor ϒ_1_ is obtained through |Ω(*S*)| multiplications and |Ω(*S*)| − 1 sums— to find *P*(*S*_0_ = *s*_0_)—and *T* − 1 products by entries of the transition model. Its time complexity is equal to *O*(|Ω(*S*)| + *T*). Finally, the algorithm in [20] has a run time of *O*(|Ω(*S*)|^2^ ⋅ *T*), plus |Ω(*S*)| − 1 additions to compute ϒ_2_. Hence, the computation of ϒ_2_ using this algorithm is the slowest of the three. Indeed, this is also the overall time complexity of the algorithm:

$$\begin{array}{c} O(|\Omega \left(S\right){|}^{2}\cdot T) \end{array}$$ (11)

We observe, in fact, that all the three factors ϒ_0_, ϒ_1_, and ϒ_2_ are independent (from a computational point of view, not with regard to probability) and they can be can be easily computed in parallel, with the last one strictly dominating the others.

Most importantly, we remark that, if the time horizon is much larger than the number of states (i.e. *T* ≫ |Ω(*S*)|), the probabilistic inference algorithm has an overall time complexity dominated by *O*(*T*). This result reveals that our algorithm—despite answering the different kind of queries we are interested in—belongs to the same time complexity class of other well-known inference algorithms for HMMs: the forward-backward algorithm for the computation of smoothed marginals distributions, and the Viterbi algorithm for the computation of the most likely sequence of hidden variables [9].

The data structures necessary to represent the c-HMM framework have moderate memory requirements: *P*(*S*_0_) has size of *O*(|*S*|), *P*(*O*_*t*_∣*S*_*t*_) of *O*(|Ω(*S*)| ⋅ |Ω(*O*)|), *P*(*S*_*t*+1_∣*S*_*t*_) of *O*(|Ω(*S*)|^2^), and *c* of *O*(|Ω(*S*)|). The input of our inference queries consists of two vectors, a trajectory of states *s*_0_, …, *s*_*T*_ and a trajectory of observations *o*_0_, …, *o*_*T*_, having size of *O*(*T*) each. The computation of ϒ_0_ requires to iteratively multiply the result of a previous product and store a single floating point value, hence, its space complexity is *O*(1). Similarly, the factor ϒ_1_ can be computed by repeatedly storing the result of successive additions and multiplications in the same memory cells and it has space complexity of *O*(1). Finally, the execution of the algorithm to find ϒ_2_ [20] demands memory of *O*(|Ω(*S*)|), again dominating the other two factors.

As a result, the memory requirements for the computation of the probability of a trajectory of states, given a trajectory of observations are:

$$\begin{array}{c} \begin{array}{c} \text{1)}\;\text{model:}\;O(|\Omega \left(S\right)|\cdot |\Omega \left(O\right)|+|\Omega \left(S\right){|}^{2})\\ \text{2)}\;\text{input:}\;O(T)\\ \text{3)}\;\text{algorithm:}\;O(|\Omega (S)|) \end{array} \end{array}$$ (12)

**Fig 2:**
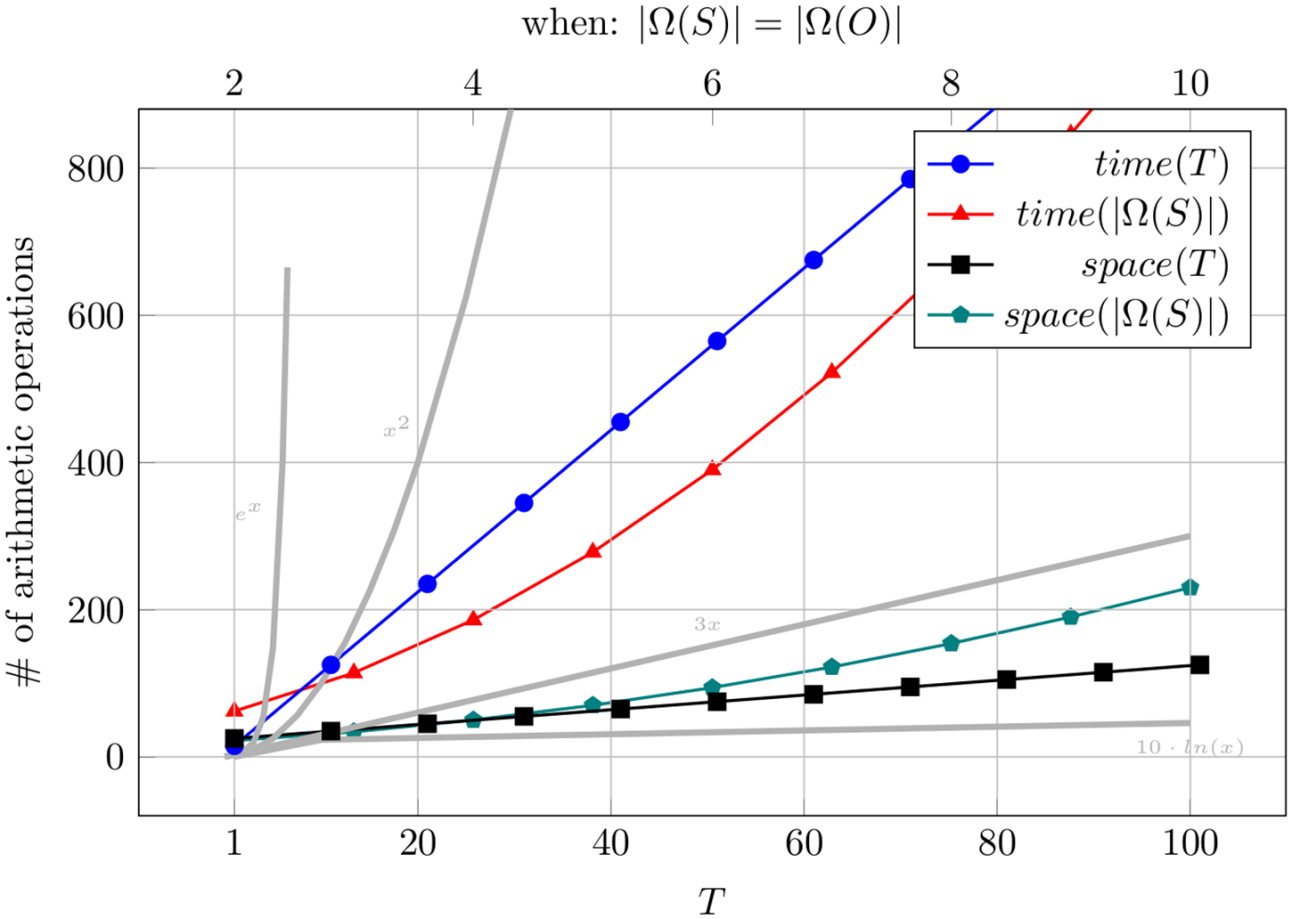
Inference complexity. Theoretical complexity growth of the proposed inference algorithm with respect to the time horizon *T* and the size of the state domain |*S*|. In the legend, *time* and *space* stand for time-complexity and space-complexity, respectively.

This also means that the space complexity of the inference algorithm itself do not depend on the time horizon *T*. In most practical cases, in which *T* ≫ |Ω(*S*)|, the memory bottleneck will be represented by the memories dedicated to the storage of the input sequences *s*_0_, …, *s*_*T*_ and *o*_0_, …, *o*_*T*_. Fig 2 shows how time and space complexity evolves with respect to the size of the inputs.

## Complexity of generic property checking

The complexity analysis in the previous section was that of an algorithm capable of computing the probability of a trajectory of states, given an assignment of the trajectory of observations. We have shown that this can be done, rather inexpensively, in time *O*(*T*): a result that makes our algorithm as good as the best state-of-the-art algorithms for exact inference in HMMs. However, probabilistic property checking in c-HMMs requires an additional step: the identification of those trajectory of states that actually enforce a certain property. The complexity of this step is, in general, property-dependent.

The number of all possible assignments of a trajectory of states, is equal to |Ω(*S*)|^*T*^. Unless a number of impossible (i.e. zero-valued) states or transitions appear in the HMM in either *P*(*S*_0_) or *P*(*S*_*t*+1_ ∣ *S*_*t*_), all these assignments will have non-null probabilities. However, it can be noted that properties are functions of (i.e. only depend on) trajectories of costs.

**Proposition 1**. *Given a finite time horizon T and a c-HMM* $\langle P\left(S_{0}\right),P\left(O_{t}|S_{t}\right),P\left(S_{t+1}|S_{t}\right),c:\Omega \left(S\right)\to R^{+}\rangle$, *the number of possible assignments of the trajectory of costs is equal or smaller than the cardinality of the set of trajectories of states*.

*Proof*. This holds true as a consequence of the properties of the function *c*: ∀*q* ∈ {*q*∣*q* = *c*(*s*) ∧ *s* ∈ Ω(*S*)}, |*c*^−1^(*q*)| ≥ 1.

On the other hand, the number of trajectories of states that share the same trajectory of costs is: $\prod_{i=1}^{T}\left|c^{-1}\left(k_{i}\right)\right|$ (where *k*_*i*_ is the cost of the state at the *i*-th time step). Let $K\subset R^{+}$ be the set of all the costs that are images of the possible assignments of *S*: *K* = {*k* ∣ *k* = *c*(*s*) ∧ *s* ∈ Ω(*S*)}, the largest *max*_*k*∈*K*_|*c*^−1^(*k*)|, the smallest the size of the search space of the trajectories of costs were the property checking actually happens. Contrarily, if *max*_*k*∈*K*_|*c*^−1^(*k*)| = 1, property checking over trajectories of costs is isomorphic to property checking over the assignments of trajectories of states.

## Bounding the probability of l-resistance

The main challenge of dealing with long time horizons *T* is that, as the number of possible trajectories grows exponentially with their length, the subset of trajectories that satisfy a certain (resilient or not) property might grow as well. This is also true and especially important for the resilient property of *l*-resistance.

**Proposition 2**. *This additional layer of complexity cannot really be circumvented, i.e., in general it is not possible to preclude the exponential growth of the number of probability values that must be evaluated to assess the probability of l-resistance*.

*Proof*. This derives from fact that *P*(*S*_1_ ≤ *l* ∧ *S*_2_ ≤ *l*|*o*_1_, *o*_2_) cannot be factorized into *P*(*S*_1_ ≤ *l*|*o*_1_, *o*_2_) ⋅ *P*(*S*_2_ ≤ *l*|*o*_1_, *o*_2_) because 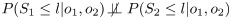, excluding the application of the principles of induction through an iterative algorithm.

Instead, we can use the algorithm detailed in S1 Appendix to compute approximated probability values of *l*-resistance that are strictly smaller—or larger—than the actual value, i.e. plausible lower and upper bounds of *P*(*S*_0_ ≤ *l* ∧ ⋯ ∧ *S*_*T*_ ≤ *l*|*o*_0_, …, *o*_*T*_). To compute a pessimistic estimate of this probability, we must construct new pseudo- transition and sensor models $\hat{P}\left(S_{t+1}|S_{t}\right),\hat{P}\left(O_{t}|S_{t}\right)$. We aggregate all the states that have cost ≤ *l* or > *l* into two macro-states *s*_≤*l*_, *s*_>*l*_ so that:

$$\begin{array}{c} \begin{array}{c} \hat{P}(s_{t+1}=s_{\leq l}|s_{t}=s_{\leq l})=\underset{\begin{array}{l} \text{i}\;\;\text{s.t.}\;\;c\left(s_{i}\right)\leq l\text{,}\\ \text{j}\;\;\text{s.t.}\;\;c\left(s_{j}\right)\leq l \end{array}}{\text{min}}P(s_{t+1}=s_{i}|s_{t}=s_{j})\\ \hat{P}(s_{t+1}=s_{\leq l}|s_{t}=s_{>l})=\underset{\begin{array}{l} \text{i}\;\;\text{s.t.}\;\;c\left(s_{i}\right)\leq l\text{,}\\ \text{j}\;\;\text{s.t.}\;\;c\left(s_{j}\right)>l \end{array}}{\text{min}}P(s_{t+1}=s_{i}|s_{t}=s_{j}) \end{array} \end{array}$$ (13)

$$\begin{array}{c} \begin{array}{c} \hat{P}(s_{t+1}=s_{>l}|s_{t}=s_{\leq l})=\underset{\begin{array}{l} \text{i}\;\;\text{s.t.}\;\;c\left(s_{i}\right)>l\text{,}\\ \text{j}\;\;\text{s.t.}\;\;c\left(s_{j}\right)\leq l \end{array}}{\text{max}}P(s_{t+1}=s_{i}|s_{t}=s_{j})\\ \hat{P}(s_{t+1}=s_{>l}|s_{t}=s_{>l})=\underset{\begin{array}{l} \text{i}\;\;\text{s.t.}\;\;c\left(s_{i}\right)>l\text{,}\\ \text{j}\;\;\text{s.t.}\;\;c\left(s_{j}\right)>l \end{array}}{\text{max}}P(s_{t+1}=s_{i}|s_{t}=s_{j}) \end{array} \end{array}$$ (14)

and, ∀*o*_*j*_ ∈ Ω(*O*), we have:

$$\begin{array}{c} \begin{array}{c} \hat{P}\left(o_{t}=o_{j}|s_{t}=s_{\leq l}\right)=\min_{i\;s.t.\;c(s_{i})\leq l}P\left(o_{t}=o_{j}|s_{t}=s_{i}\right)\\ \hat{P}\left(o_{t}=o_{j}|s_{t}=s_{>l}\right)=\max_{i\;s.t.\;c(s_{i})>l}P\left(o_{t}=o_{j}|s_{t}=s_{i}\right) \end{array} \end{array}$$ (15)

Plugging this new models in our algorithm, one can compute a lower bound, i.e. a value smaller or equal, for the probability of the trajectory being *l*-resistant. Similarly, one can compute an upper bound for the probability of resistance, using the pseudo- transition and sensor models of *s*_≤*l*_ obtained by swapping the min and max operators in the definitions above. Therefore, the new model and our algorithm allow to compute, with time complexity that is linear with the length of the trajectory, an interval [*P*_*low*_, *P*_*up*_] that certainly contains the *l*-resistance probability *P*(*S*_0_ ≤ *l* ∧ ⋯ ∧ *S*_*T*_ ≤ *l*|*o*_0_, …, *o*_*T*_).

Approximate inference methodologies are widely used in the context of Bayesian networks, dynamic Bayesian networks, and probabilistic graphical models in general. Unlike the upper and lower bound approximation proposed above, however, most approximate inference methods for probabilistic graphical models are based on repeated randomized sampling, also known as Monte Carlo methods or algorithms [9]. Rejection, likelihood weighting, Markov chain, and Gibbs sampling are all approaches for Bayesian networks inference that, with certain limitations and adjustments, can be adapted to the family of dynamic nets [9]—to which HMMs belong. In many real-world applications, approximate inference becomes necessary when the complexity of the model renders exact inference intractable. To the contrary, we observe that the complexity of models and inference in this work is always kept within the realm of the exactly tractable—with the issue of unrestrained growth being circumscribed to the number of state trajectories satisfying any specific property. Sampling, therefore, could be used orthogonally to the proposed exact inference, as a tool to explore the space of state trajectories (see S1 Appendix).

## Application scenarios

To demonstrate the potential of the proposed methodology, we apply both its modelling and probabilistic inference facets to four practical scenarios. These examples serve to demonstrate that resilience and the resilient properties have a prominent role in several different domains. Moreover, they show that, in the “random world” [1] we frequently encounter environments that have non-deterministic dynamics and are observed through noisy, imperfect, or broken sensors (i.e. partial observability). The first two qualitative examples are inspired by the domains of disaster management and macroeconomics. The third and fourth example are drawn from the fields of self-adaptive computing for aerospace applications and swarm robotics, respectively, and they are used to evaluate the quantitative aspects of the proposed approach as well.

### Disaster management

**Fig 3:**
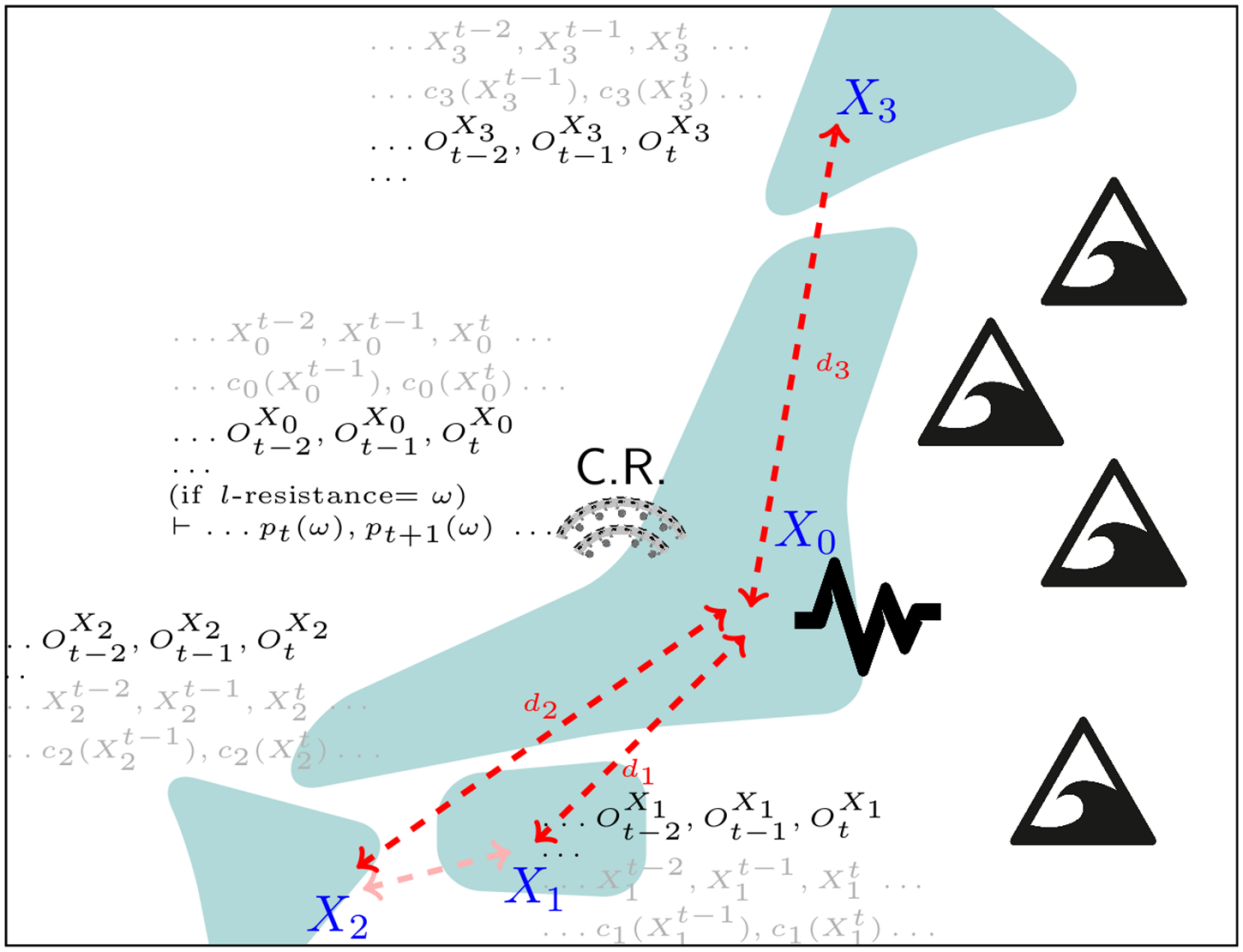
Disaster management. The four island archipelago modelled in the first application scenario. For each island, the image shows its geographical distribution, the evolving state, cost, and (partial) observation from the point of view of the control room. This figure is similar but not identical to the one in the original submission and it is for illustrative purposes only.

When dangerous disruptive events occur, proper disaster management is crucial to protect human lives and minimize casualties [21]. Effective disaster management cannot be decoupled from good modelling and decision making strategies [22]. In our first application scenario, we model a four islands archipelago *X*_0_, *X*_1_, *X*_2_, *X*_3_ (see Fig 3) that can be affected by three different level of alert $D\left(X_{i}\right)=a_{0},a_{1},a_{2}$—from “no intervention needed” (*a*_0_) to “emergency” (*a*_2_), passing by “some intervention needed” (*a*_1_). In this example, |Ω(*S*)| = 3^4^ = 81. To take into account the different speeds at which alerts escalate and get re-absorbed on each islands, we define four transition models ∀*i* ∈ [0, 3], *P*_*i*_({*X*_*i*_}_*t*+1_|{*X*_*i*_}_*t*_) and construct the overall probabilistic dynamics as *P*(*S*_*t*+1_|*S*_*t*_) = *P*({*X*_0_, *X*_1_, *X*_2_, *X*_3_}_*t*+1_|{*X*_0_, *X*_1_, *X*_2_, *X*_3_}_*t*_) = ∏_*i*_ *P*_*i*_(*X*_*t*+1_|*X*_*t*_) (this also implies that the alert status as independent from one another). We assume that the observations domain is isomorphic to that of the states Ω(*O*) = Ω(*S*), that an “emergency control centre” resides on the *j*-th island, and that the reliability of an observation decays exponentially with the distance it has to travel (this is, for example, the case of a multi-hop communication network with constant packet drop between any two nodes but one could also choose to plug-in any of the more sophisticated probabilistic models found in the literature [23]). Having defined the observation of the *i*-th island status as $O^{X_{i}}$ and its distance from the control centre as *d*_*i*_, then $P_{i}\left(O_{t}^{X_{i}}=a_{p}|X_{t}=a_{q}\right)=e^{-d_{i}}\;\text{if}\;a_{p}=a_{q}\text{,}\;\text{and}\;\left(1-e^{-d_{i}}\right)/(|\Omega \left(O\right)\left|-1\right)\;\text{otherwise}$. The overall observation model is defined as $P\left(O_{t}|S_{t}\right)=P\left({\left\{O^{X_{0}},O^{X_{1}},O^{X_{2}},O^{X_{3}}\right\}}_{t}|{\left\{X_{0},X_{1},X_{2},X_{3}\right\}}_{t}\right)=\prod_{i}P_{i}\left(O_{t}^{X_{i}}|X_{t}\right)$. Functions $c_{i}\left(X_{i}\right):D\left(X_{i}\right)\to N$ state how many resources, e.g. the number of search and rescue teams, have to be sent to the *i*-th island, depending on its alert status. The cost function $c\left(S\right):\Omega \left(S\right)\to N^{4}=\sum_{i}c_{i}$ reveals how many resources are required to cope with each situation in the state domain.

The numerical values in the transition and the sensor model can be found or improved upon used historical data and expert knowledge. The emergency control centre can use them to look at the stream of information about the alert status (or their prediction through the transition model *P*(*S*_*t*+1_|*S*_*t*_)). Performing inference on the system model allows to answer different queries of interest. If a limited number of search and rescue teams are present on the archipelago, computing the probability of the *l*-resistance property to hold true and making sure that it is above a desirable threshold (e.g. *p*(*ϕ*(*resistance*, *l*)) ≥ 0.95), ensures that *l* is the correct number of resources to deal with the potential emergencies—should the resistance probability drop, more resources would be necessary. Furthermore, if the archipelago can temporarily recall an additional *q* resources (e.g. from a national guard), the *p* parameter for which the probability of 〈*p*, *q* + *l*〉-recoverability is above a safe limit will tell for how many time steps (days or months) those extra resources should be mobilized (and therefore paid, quartered, etc.).

### Macroeconomics

Probabilistic and statistical models are already widely exploited tools in the fields in economics and finance—the latter especially. A notable example being the research on the expected return and risk of efficient portfolios by Harry Markowitz [24]. The deterministic modelling approach of traditional macroeconomics, on the other hand, has come to be questioned over the last decade by the crisis of 2008 and the growing prominence of experimental and behavioural economics [25]. Reckoning the existence of yet many unknowns in modern macroeconomics, probability theory only seems the natural development direction for models that need to be able to account for the uncertainties of this domain and the irrationality of human behaviours.

Applying the proposed modelling to the context of macroeconomics gives us a tool to derive valuable insights this world. Assessing the resilience of a macroeconomic system is important for multiple reasons: smoothly running economics guarantee the development, stability, and fairness of our societies. The 2008 housing market crisis proved that existing models are not enough to protect us from rare, non-directly observable, and counterintuitively correlated events [26]. The argument that macroeconomics should be revisited to deal with the uncertainty of the real world is not new [27] and statistical model for certain phenomena have been proposed [28]. Existing research can be leveraged by our approach by simply verifying that the Markov property is enforced *P*(*S*_*t*+1_|*S*_*t*_). The general consensus on the yet incomplete understanding of macroeconomics lends itself perfectly to a partially-observable modelling approach *P*(*O*_*t*_|*S*_*t*_). As economists are well aware of the limitations of existing models, they often rely on stress tests [29] to evaluate the resilience of financial institutions [30]. Stress tests are experimental tools that go beyond statistical analysis but, for which, statistical meta-analyses exist [31] and can be used to construct the observation model required by our approach. Intuitively, the cost function of the c-HMM describing this scenario will tell which amount of money (in cash, deposits, or bonds) a government would need to prevent a default in a certain state. Governments, banks, and investment funds typically monitor time horizons of 5, 10 (sometimes 15, 20) years. In this context, the *f*-functionality property represents the amount of funding that has be made available, on average, across multiple year budgets. The 〈*p*, *q*〉-recoverability property tells how far into debt a government would have to go to recover from a crisis within a fixed timeframe.

### Self-adaptive computing

Self-adaptive computers possess *ad hoc* capabilities—e.g. sensors, actuators, and decision making loops [32]—that allow them to express autonomous behaviours. Because they do not require the supervision of a human operator, these systems are especially suitable for critical, advanced applications such as space systems and robotic exploration. An autonomous computer and a resilient ecological system share several properties, for example the ability to self-protect and self-heal [33] and assessing the resilience of the first is of primary importance both at design and run time. Previous research [34–36] proved that probabilistic models have the potential to enable autonomous computing systems. We now demonstrate how they can be exploited for the analysis of their resilience.

The ArduSat Payload Processor Module (ASPPM) carried by the 1U CubeSat [37] ArduSat-1 consists of one supervisor processor and 16 processing elements (PEs), and it is the ideal platform for a modular, redundant autonomous on-board computer (OBC). The resilience of the OBC of a spacecraft is typically enforced through the software and/or hardware replication of its essential functionalities: (1) housekeeping (C&DH), i.e. all the software tasks contributing to the monitoring of the satellites status and the correct execution of its routine functions; (2) the processing of the data collected by the payload of the satellite while performing its mission (Mission), e.g. running a classification algorithm over the images captured by a camera [38]; and (3) the attitude control algorithm (ACDS), responsible for the proper orientation of the satellite with respect to Earth and its targets, through the computation of the control signals of the satellite actuators (e.g. reaction wheels).

Because of the harsh toll posed by space weather (solar wind, cosmic rays) on electronics, each of the processing elements ∀*i* ∈ [0, |*PE*| − 1], *pe*^*i*^ in the set *PE* can find itself in one of three states: *pe*^*i*^ ∈ {*w*, *t*, *p*}, that is, correct operation *w*, experiencing a transient fault state *t*, or permanent failure *p*. A stochastic transition model describes the ageing of a PE [34] and it is parameterized by the impact rate of particle radiation *r* and the mean time to failure *MTTF* of a PE. These parameters are responsible for transient and permanent faults, respectively. *r* is strongly orbit-dependent and is computed with the aid of radiation models such as Creme96 and SPENVIS [39, 40]. Assuming independence among the evolution of the PEs and defining the state of the system as *S* = {*pe*^*i*^ *s*.*t*. *i* ∈ [0, |*PE*| − 1]}, we can generalize the transition model [34] as follows (where *W* is the duration of a time step):

$$\begin{array}{c} P\left(S_{t+1}|S_{t}\right)=\prod_{i=0}^{|PE|-1}P\left(pe_{t+1}|pe_{t}\right)=\{\begin{array}{c} \text{if}\;pe_{t}^{i}=\left(w||t\right)\text{:}\;P\left(pe_{t+1}^{i}\right)=\left\langle \frac{1-r}{e^{W/MTTF}},\frac{r}{e^{W/MTTF}},1-\frac{1}{e^{W/MTTF}}\right\rangle \\ \text{if}\;pe_{t}^{i}=p\text{:}\;P\left(pe_{t+1}^{i}\right)=\left\langle 0,0,1\right\rangle \end{array} \end{array}$$ (16)

In the case of ArduSat-1, the observers of the resilient system are the ASPPM’s on board supervisor ATmega2561 microcontroller and the external NanoMind A712C flight control computer. Observations of each PE, however, are not perfect for two reasons: (1) errors can slip into the observers too; and (2) transient and permanent faults are, *a priori*, indistinguishable. Our approach seamlessly models these kinds of observations with a framework that accounts for both “partial” (in modal logic, ¬□(Ω(*S*) = Ω(*O*))) and probabilistic observability. Having defined the observation of each PE as working or faulty, $O^{pe_{i}}\in \left\{w,f\right\}$, and the system observation as the set of observation of all PEs, $O=\{O^{pe^{i}}\;s.t.\;i\in \left[0,|PE\right|-1]\}$, we can use any suitable memoryless probability distributions for the sensor model [34] (with false positive and false negative rates of *p*^*fp*^, *p*^*fn*^):

$$\begin{array}{c} \begin{array}{c} P\left(O_{t}|S_{t}\right)=\prod_{i=0}^{|PE|-1}P\left(O_{t}^{pe^{i}}|pe_{t}^{i}\right)=\{\begin{array}{c} \text{if}\;pe_{t}^{i}=w\text{:}\;P\left(O_{t}^{pe^{i}}\right)=\left\langle 1-p^{fp},p^{fp}\right\rangle \\ \text{if}\;pe_{t}^{i}=\left(t||p\right)\text{:}\;P\left(O_{t}^{pe^{i}}\right)=\left\langle p^{fn},1-p^{fn}\right\rangle \end{array} \end{array} \end{array}$$ (17)

The cost function expresses the utility [9] of a configuration, that is, the scientific data throughput (e.g. in MBytes per orbit or per day) that a certain state configuration puts on the downlink of the satellite’s telecommunication system. In general, this data throughput is a function of the state of the ASPPM *s* ∈ *S*, the orbit of the satellite *ξ* ∈ *Ξ*, and number/position of ground stations *ψ* ∈ *Ψ*: $ST\left(s,\xi ,\psi \right):S\times \Xi \times \Psi \to R^{+}$. For a given low-Earth orbit $\bar{\xi }$ with a 400km altitude and 51° inclination, and a single ground station $\bar{\psi }$ in North America, we write $c^{\bar{\xi },\bar{\psi }}(S)$ as the cost function of the ASPPM state as:

$$\begin{array}{c} c^{\bar{\xi },\bar{\psi }}\left(S\right)=ST\left(S,\bar{\xi },\bar{\psi }\right)=\{\begin{array}{c} \ldots \\ 3.7MB/day\;\text{if}\;S^{i-1}=\left\langle ⌀;⌀\right\rangle ;\;map:\;\left\langle c\&dh\mapsto pe^{9,13:14};mission\mapsto pe^{2:4,6:8};acds\mapsto pe^{10:12,15:16}\right\rangle \\ 2.0MB/day\;\text{if}\;S^{i}=\left\langle ⌀;4\right\rangle ;\;map:\;\left\langle c\&dh\mapsto pe^{9,13:14};mission\mapsto pe^{2:3,6:7};acds\mapsto pe^{10:12,15:16}\right\rangle \\ 1.3MB/day\;\text{if}\;S^{i+1}=\left\langle ⌀;4,12\right\rangle ;\;map:\;\left\langle c\&dh\mapsto pe^{9,13};mission\mapsto pe^{3,7};acds\mapsto pe^{10:11,15}\right\rangle \\ \ldots \end{array} \end{array}$$ (18)

**Fig 4:**
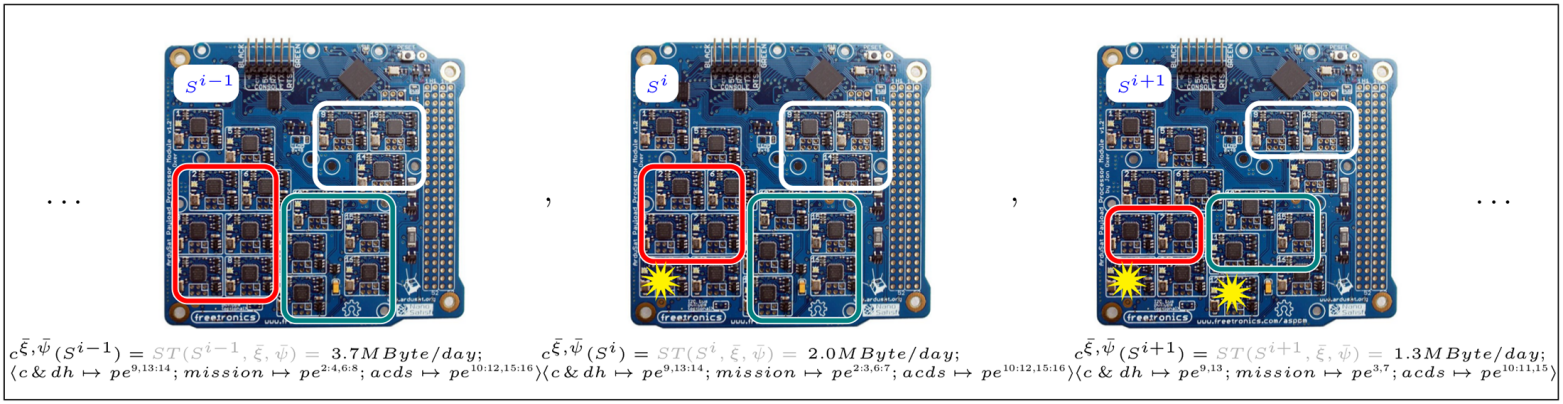
Self-adaptive computing. A visual representation of three of the possible “software task”-to-“hardware resource” mappings in the state space of the 1U CubeSat’s Arduino-based ASPPM from the third application scenario, as presented in Eq 18.

In Eq 18, the shortcut 〈⌀; 4〉 is used to indicate a state in which no PE is experiencing a transient fault, and *pe*^4^ is permanently faulty (all other PEs are assumed to work correctly); *map* specifies how the software tasks are mapped to the PEs in any given state. Fig 4 offers a visual reference of the subset of these mappings, as in Eq 18.

To discover meaningful semantics associated to the resilient properties, we introduce a helper (cost) function $\hat{c}\left(S\right)=c-c\left(S\right)$, where $\bar{c}$ is the theoretical maximum throughput attainable by the satellite. Using $\hat{c}\left(S\right)$, computing the probability distributions of *f*-functionality and *l*-resistance reveals the expected and worst-case data throughput sent to Earth, respectively. The property of 〈*p*, *q*〉-recoverability can help quantify the loss of scientific data in the case of drops in the throughput (due to faults or reconfiguration of the system). The advantage of using the algorithm proposed in this work (see S1 Appendix) to assess these properties is the ability to maintain the computation within reasonable time limits, even for relatively long traces and complex models. In a search space of 3^16^ states, 2^16^ possible observations, and time horizons of 10, 100, or 1000 steps, the proposed approach requires a number of arithmetic operations in the order of 10^13−15^ to compute the probability of a state trajectory. The same problem would simply be intractable by any other algorithm that requires to evaluate the 10^75^ entries in the conditional joint probability distribution (CJPD). Because of the exponential growth of the CJPD, the savings are remarkable (order of 10^59^) even for properties that are satisfied by a large (e.g. 30%) fraction of the possible state trajectories.

The potential of self-aware computing is not limited to satellites. Studying the challenges of Mars rover operations, Gaines *et al.* [41] outlined a model of seven factors impacting productivity. Among non-human factors, they identified the reliability of the uplink/downlink as a cause for “deferred” sols—i.e., Martian solar days in which the campaign objectives have to be postponed to address unexpected issues. Indeed, they suggest “state-aware health assessment” as one of the capabilities that shall be developed in future missions to mitigate this problem.

For example, NASA and JPL’s most recent Mars rover, Curiosity, is able to perform ∼5h/sol of tactical science activities [42]. This is due to the fact that direct-to-Earth communication is limited—by power and orbital constraints—to a few hours/day at data rates of 0.5 to 32kb/s. Therefore, most transmissions are relayed by two sun-synchronous orbiters—Mars Reconnaissance Orbiter, at up to 2Mb/s, and Odyssey, at 128 or 256kb/s. Each of the orbiters passes over the rover, every sol, for a 8’-window while they can both transmit to Earth for ∼16h/day. Commands are uploaded to the rover every sol during an overnight orbiter pass (or direct-from-Earth at local midmorning). Data that are necessary to plan the activities of the following sol are then returned via an orbiter telecom pass in the midafternoon. Non-essential information is stored and returned during the following overnight orbiter pass [42]. As a consequence, if the rover fails to send the required information during the correct orbiter pass, the tactical team might not be able to plan the activities for the following sol. This is an issue that will aggravate in the near future, as the current fleet of sun-synchronous orbiters is replaced with non-sun-synchronous orbiters [41].

As originally planned, Curiosity’s primary mission spanned over 669 sols. Accounting for (i) a commissioning phase of 90 sols, (ii) 30 sols of solar conjunction, (iii) 10 sols for maintenance and updates, (iv) a 20% of “not commandable” sols due to Earth-Mars phasing, and (v) a 25% of “non-productive” sols “due to unforeseen shortfalls in mission resources […] or communication problems” [42], the rover was left with ∼300 sols to explore the vicinity of the Gale crater, traverse ∼18km, and collect ∼11 samples. With hindsight, the 25% estimate of “non-productive” sols proved to be rather conservative: the study in [41] observes that tactical activities were only deferred in 3 out of 19 (16%) sols during 2014’s Pahrump Hills campaign and in 1 out of 24 (4%) sols during 2015’s Artist’s Drive. Yet, self-aware computing might have the potential to further improve performance, e.g., with the implementation of a decision support system (DSS) on top of the self-assessment framework described in this work.

Having associated probability values to the data throughput of a computing system (through a model as the one in Eqs 16 to 18), a binary classification/decision system would autonomously choose whether to use the overnight orbiter pass to (i) transmit the non-essential information (the default behaviour) or (ii) re-transmit the data required for tactical planning (when it believes that the previous transmission failed) and prevent unproductive sols. The sensitivity and specificity of the classifier are affected by several factors (including the noisiness of the on-board sensors and the time horizon of the assessment algorithm). However, even assuming relatively weak performance (e.g., sensitivity and specificity of 0.8) and the conservative “deferred sol” incidence of [41], this DSS could reduce the number of unproductive sols by 3.2–12.8%. Over the course of the >1600 sols spent by Curiosity on Mars, it means 50-to-200 extra sols of science activities, equivalent to 3-to-12 extra kilometres and 2-to-7 additional samples.

### Swarm robotics

**Fig 5:**
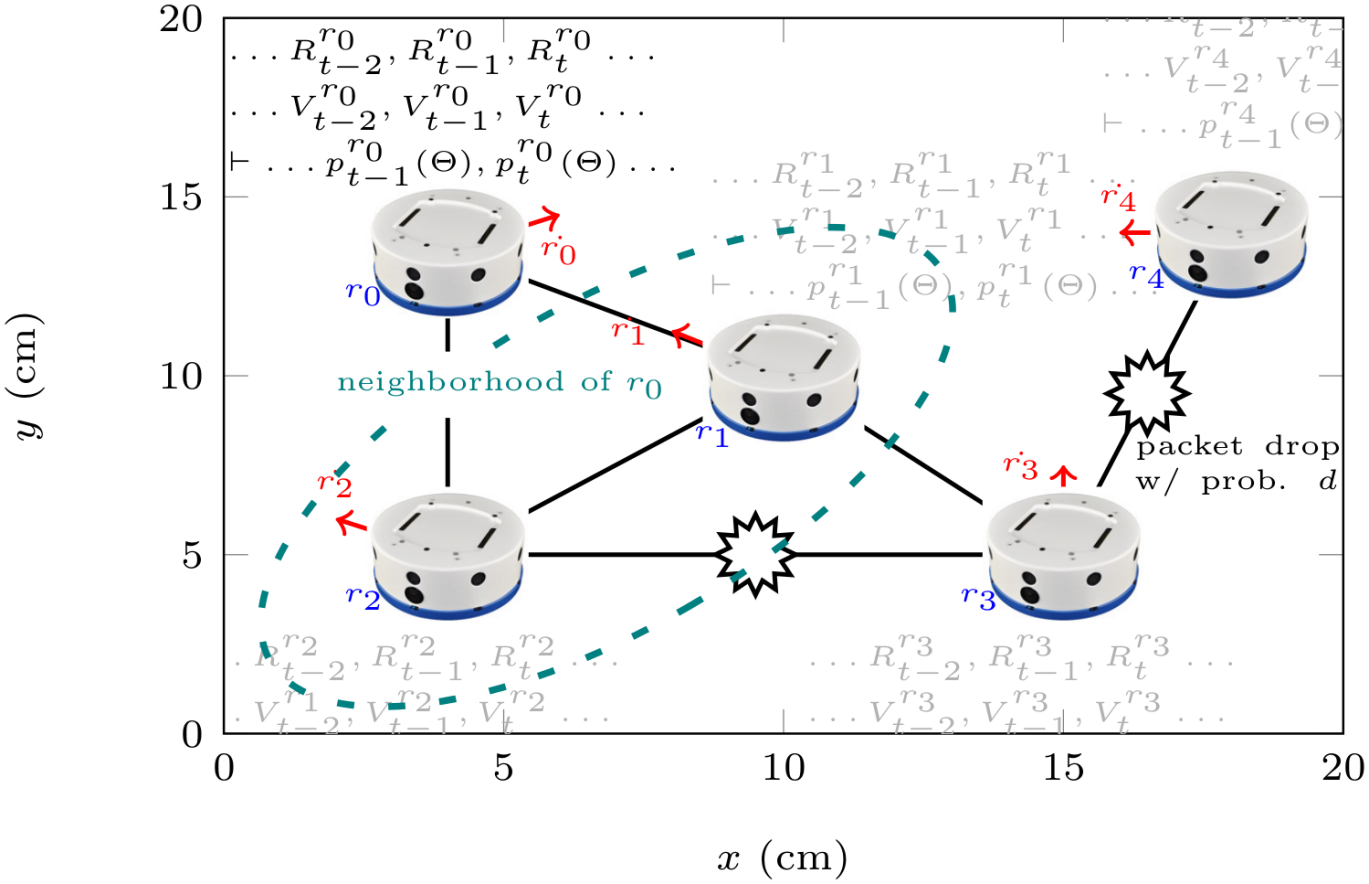
Swarm robotics. A robotic swarm, as described in the fourth application scenario. Each robot possesses a position, velocity, state (the number of its neighbors), and a partial observation (of its neighborhood) evolving over time. The inference algorithm is executed locally to assess the probability of losing connectivity with respect to the rest of the swarm at each time step.

As many-robot systems, or robot swarms, become more and more pervasive, researchers must devise new, efficient ways to control and coordinate them [43]. In the fourth practical scenario, we test our framework in the context of the networked multi-robot system of Fig 5, where robots move independently and have a limited communication range. We implemented a simulator for the robots’ movement and communication model, the proposed algorithm, and an alternative reference approach based on the computation of the conditional joint probability distribution. We remark that computing the CJPD is already a more efficient approach than blindly expanding the entire joint probability distribution of a c-HMM. We analyze the scenario with two examples: a small one with 4 robots in a 20cm by 20cm arena and a large example with 20 robots in a 40cm by 40cm area. In both examples, robots have a diameter of 2cm (similarly to Kilobots [44]), move on independent random walks at a speed of 2cm/s, and have a communication range of 10cm.

As a transition model, we use the conditional probability distribution that describes the way in which the number of neighbors *R* of a robot evolves over a time step of 1s: *P*(*R*_*t*+1_|*R*_*t*_). To empirically derive this model, in both examples, we performed 30 random-walk simulations of 10′ each, with the positions of the robots randomly initialized. For the sensor model, we assume that communication links between neighbors can be temporarily broken with probability *d* = 0.1. As a consequence, the sensor model that describes the number of robots *V* that are actually visible to a robot with *R* neighbors follows the binomial distribution: *P*(*V*_*t*_|*R*_*t*_) = *P*(*X* = *V*_*t*_) with *X* ∼ *B*(*R*_*t*_, *d*). We want to assess the probability of the following two properties:

- Λ (*l*-resistance), given a series of observations over a time horizon *T* varying from 4 to 6, property Λ guarantees that a robot always maintained more than *l* neighbors. The *l* parameter is set to 2 in the small example and 10 in the large one.
- Θ: given a series of observations over a time horizon *T* varying from 4 to 6, property Θ says that a robot lost connectivity (i.e. found itself in a position with zero neighbors) precisely during the last timestep—and not before.

Table 1 reports the results of specific experiments, taking typical series of observations as inputs. It is worth noting that, because the proposed one is an exact approach, the obtained probability values are identical w.r.t. those extracted from the CJPD, while—from the results of the experiments—it emerges that the computational time is reduced by a factor ranging between 10^2^ and 10^4^. In the 4 robots/6 steps time horizon case, for example, the computational time of the probability of property Λ is lowered from >1000s to ∼0.01-0.1s.

**Table 1:** Computational time savings yield by the proposed approach.

Observation Trajectory *TO*	# of Robots	*p* of Λ	Computation time (s)	
			CJPD	Proposed
[2, 2, 1, 2]	4	0.77167	3.022	0.003
[10, 10, 8, 10]	20	0.40485	n/a	1.964
[2, 2, 2, 2, 1, 2]	4	0.77033	1027.0	0.018
[10, 10, 10, 10, 8, 10]	20	0.38312	n/a	271.51
Observation Trajectory *TO*	# of Robots	*p* of Θ	Computation time (s)	
			CJPD	Proposed
[2, 2, 1, 0]	4	0.52712	3.059	0.005
[2, 2, 1, 0]	20	0.54640	n/a	1.354
[2, 2, 2, 2, 1, 0]	4	0.52727	1025.5	0.063
[2, 2, 2, 2, 1, 0]	20	0.53958	n/a	698.14

Experimental results from the fourth application scenario, describing a robot in the small or large swarm trying to assess the probability of properties Λ and Θ using only local and the—possibly faulty—observations of its neighborhood.

**Fig 6:**
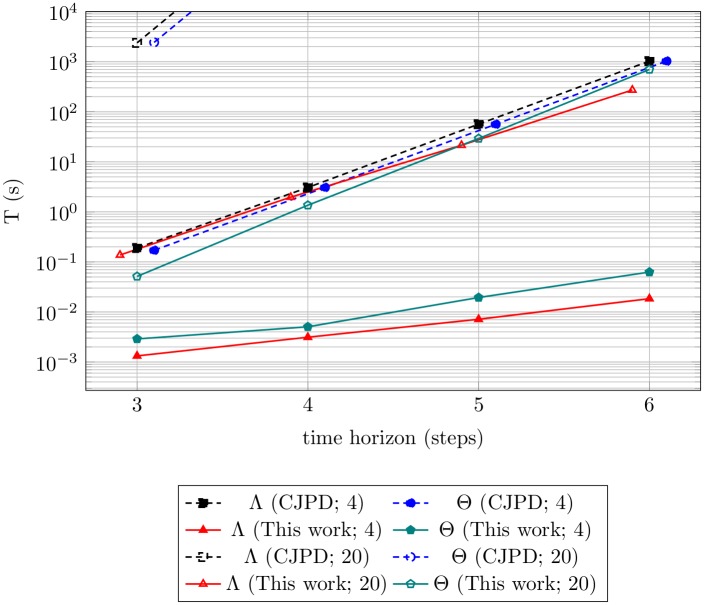
Complexity of assessing swarm robotics’ properties. Experimental assessment of the time complexity and comparison of the scalability of the computational time of different queries for property Λ and property Θ through the algorithm proposed in this work versus expanding the conditional join probability distribution, in the 4 robots and 20 robots scenarios.

Fig 6 compares the time delay of the proposed approach and that required by the computation of the CJPD. For large scenarios, the CJPD delay rapidly gets off the chart. The proposed algorithm, instead, allows to deal with 20 robots with a comparable, but smaller, delay than the one required by the computation of the CJPD in the 4 robots scenario. In particular, we observe that the advantage of the proposed approach over the use of the CJPD actually increases with the length of the time horizon and the number of robots in the scenario. Being able to perform exact inference in only seconds in large scenarios, accounting for tens of robots, this approach can effectively be implemented in several practical multi-robot applications, such as target tracking, area coverage, or task allocation. Unlike previous work on resilient robot formations and partially-observable robot swarms [16], the proposed approach does not limit the movement of the robots into configurations whose resilience can be established *a priori* but rather it allows the *a posteriori* assessment of resilience in a distributed fashion.

## Discussion

Summing up our work, we adapted the CBS/DS-based formalization of resilience given by Schwind *et al.* [17] (composed of the resilient properties of resistance, functionality, and recoverability) to the timed probabilistic framework of hidden Markov models. To do so, we defined the extended framework of c-HMMs. In S1 Appendix, we outline a state-of-the-art inference algorithm able to answer the queries required for the probabilistic property checking of resilience over this model. Furthermore, we studied the space- and time- complexity of this inference algorithm as well as those of property checking.

We demonstrated the practical applicability of our approach in four qualitative and quantitative scenarios of growing technical complexity. In our experimental evaluation, we implemented the algorithm in the Matlab-compatible scripting language GNU Octave (see the Additional Information for the supplementary materials) and tested it in the autonomous multi-processor computing system of a nano-satellite and in a multi-robot scenario to answer queries about the robots’ connectivity. The experimental results show that, even in small domains, the proposed approach is approximately (1) four orders of magnitude faster than expanding the full conditional joint probability distribution. Furthermore, the scenarios revealed that the proposed approach is capable of (2) modelling partial observability in a way that deterministic models cannot grasp and (3) leading to insights about resilience that would be, otherwise, concealed—e.g. the link between the extra resources required to probabilistically ensure 〈*p*, *q*〉-recoverability and the tightness of the associated deadline.

Looking forward, the opportunities for the further development of this work reside in the possible extensions of both its framework and the inference methodology. Despite having been able to bound the space and time complexity of each trajectory’s inference query, an existing limitation of our approach is that the number of trajectories satisfying any given property can greatly vary (depending on the nature of the property itself). In this spirit, we focussed here on a concise set of property but we recognize that, to widen applicability, approximate inference methods (for example, those based on sampling) should be investigated further. To improve the general applicability of our approach, another further step is an inference algorithm capable of dealing with missing data in the trajectory of observations. Moreover, the c-HMM framework has the potential to be enriched with the ability to perform learning, decision making, and planning—insights can be drawn from the existing frameworks of machine learning, decision networks, and MDPs.

## Supporting information

S1 Appendix Methods. The appendix explains in greater detail how we translated the concepts and definition from the section on resilience and resilient properties into a dynamic probabilistic model. It also discusses the differences between the three most common inference algorithms for hidden Markov models and the inference query used in this work (the one analyzed in the section on complexity).
(PDF)

S1 Fig Critical threshold of parametric properties. Probability distribution of the parametric resilient properties in a template scenario where ∀*s*, *c*(*s*) ∈ [0, …, 4]. The discontinuities reveal the potentially critical thresholds for different properties.
(TIF)
