# Supplementary material for: Assessing the resilience of stochastic dynamic systems under partial observability
Source: PLoS One. 2018 Aug 23;13(8):e0202337. doi: 10.1371/journal.pone.0202337 (PMC6107160; doi:10.1371/journal.pone.0202337)
Supplement: S1 Appendix — The appendix explains in greater detail how we translated the concepts and definition from the section on resilience and resilient properties into a dynamic probabilistic model. It also discusses the differences between the three most common inference algorithms for hidden Markov models and the inference query used in this work (the one analyzed in the section on complexity). (PDF) [file pone.0202337.s001.pdf]

S1 Appendix. Methods.

In the section on resilience, resilient properties, and probabilistic models we have explored the concepts of l-resistance, f-functionality, $\langle p, q \rangle$-recoverability, $\langle z, r \rangle$-resilience, and hidden Markov models. We explained that the traditional HMM framework requires to become a c-HMM in order to support the formal definition of resilience given by Schwind *et al.* [1]:

$$c\text{-}HMM = \langle P(S_0), P(O_t \mid S_t), P(S_{t+1} \mid S_t), c : \Omega(S) \rightarrow \mathbb{R}^+ \rangle \quad (1)$$

c-HMMs extend the HMM framework with a static cost function c, defined over the domain Ω of its (random) state variable S, and taking positive values in $\mathbb{R}$. We use the single discrete state variable S of a HMM as a way to represent, by enumeration, the state configuration. This information, in the constraint-based systems $CBSs$, was encoded using the set of variables $\mathbf{X}$, and assignment ς. On top of the c-HMM framework, we can define the random variables associated to trajectory of states, TS, and trajectory of observations, TO, and show how to compute their (conditioned and unconditioned) probability distributions. All the concepts presented in this section are implemented and evaluated using the open-source Matlab-compatible GNU Octave language.

States, observations, costs, and trajectories

In the SR-model, the definition of the resilient properties was based on the concepts of SSTs and their corresponding sequences of costs. In c-HMMs, we have similar constructs for states and costs, as well as observations. The main difference is that these concepts are now built on top of random variables [2] and, therefore, they also can be associated with probability distributions. Given a c-HMM and a finite time horizon T, we define its trajectory of states TS as the sequence of state variables $S_i \;\; \forall i \in \{1, \dots, T\}$. This can be rewritten as: $TS := S_0, S_1, \dots, S_T$.

S is a random variable and, therefore, TS is also a random variable. The number of possible assignments of TS grows exponentially with the time horizon: $|\Omega(TS)| = |\Omega(S)|^T$. Because the mapping provided by the cost function c is purely

deterministic, each assignment of TS is unambiguously associated with a trajectory of costs $tc = c(s_0), c(s_1), \ldots, c(s_T)$ and TC is a random variable with $|\Omega(TC)| \leq |\Omega(TS)| = |\Omega(S)|^T$.

Similar considerations are also valid for trajectories of observations $TO := O_0, O_1, \ldots, O_T$ and their possible assignments $to := o_0, o_1, \ldots, o_T$. If neither the transition model nor the sensor model contain probability values of 0, all possible sequences of states can potentially occur and produce any one of the sequences of observations. Therefore, the number of all possible configurations—i.e. entries in the joint probability distribution (JPD)—of a c-HMM is:

$$(|\Omega(S)| \cdot |\Omega(O)|)^T \tag{2}$$

This number, in principle, represents the maximum (worst-case) complexity of performing inference on our model. However, as we explained in the section on complexity, when dealing with real-world environments and the resilient properties, we are only interested in a very specific type of inference queries. That is, those queries that can return one of the $|S|^T$ probability values of the conditional probability distribution of TS with respect to a given to:

$$P(TS \mid to) = P(S_0, S_1, \ldots, S_T \mid o_0, o_1, \ldots, o_T) \tag{3}$$

This is due to the fact that: (1) the state variable S is also called the "hidden" variable as it is, in practice, never directly observable (making the actual ts taken by TS unknown); and (2) the values taken by the observation variable O are, in most cases, the one piece of *partial/imperfect* information that we can always access.

From the probability of cost trajectories to the probability of properties

We have seen that the resilient properties—once their parameters are fixed—can be considered as boolean attributes of sequences of costs associated to SSTs. In the context of the c-HMM framework, we say that an assignment of the trajectory of states $ts = s_0, s_1, \ldots$ enforces the property ϕ if and only if its corresponding trajectory of

costs $tc = c(s_0), c(s_1), \dots$ is satisfies the definition of that property, i.e. $\phi(c(s_0), c(s_1), \dots) = true$. Computing the probability distribution of parametric properties $\phi(k)$, such as resistance and functionality, with respect to their parameters, can provide valuable insights, as shown in **S1 Fig**: a rapid drop in the probability distribution might suggest the existence of a threshold cost that is unlikely to be overcome. The probability of a property $P(\phi)$ is equal to the sum of the probabilities of all the distinct trajectories of costs in which ϕ holds:

$$P(\phi) = \sum_{\forall i \in \{i | \phi(tc_i) = true\}} P(tc_i) \tag{4}$$

S1 Fig. Critical thresholds of parametric properties. Probability distribution of the parametric resilient properties in a template scenario where $\forall s,\ c(s) \in [0, \dots, 4]$. The discontinuities reveal the potentially critical thresholds for different properties.

In turn, the probability of a fixed assignment of the trajectory of costs tc is equal to the sum of the probabilities of all the distinct trajectories of states that are mapped to tc by the cost function c. To simplify the notation, we will also use $C(ts)$ to indicate the trajectory $tc = c(s_0), c(s_1), \dots$ resulting from the application of the cost function c to the assignment ts.

$$P(tc) = \sum_{\forall i \in \{i | C(ts_i) = tc\}} P(ts_i) \tag{5}$$

Plugging Eq 5 into Eq 4, one can compute the probability of a property ϕ as a function of the probabilities of distinct assignments of the trajectory of states (Eq 6). We observe that all the possible assignments of TS are "distinct" by definition, even if many of them could be mapped by c to identical trajectories of costs.

$$P(\phi) = \sum_{\forall i \in \{i | \phi(tc_i) = true\}} \sum_{\forall j \in \{j | C(ts_j) = tc_i\}} P(ts_j) \tag{6}$$

Having assumed to be able to observe the system by its trajectory of observations TO, we are then interested in computing the conditional distribution of ϕ with respect to the assignment to of TO. To do so, we start from Eq 6 and we re-write it with the

addition of conditioning on both sides by to:

$$P(\phi \mid to) = \sum_{\forall i \in \{i \mid \phi(tc_i) = true\}} \sum_{\forall j \in \{j \mid C(ts_j) = tc_i\}} P(ts_j \mid to) \tag{7}$$

Eq 7 shows that computing the probability of a property ϕ, given an assignment of the trajectory of observations to, consists of two different subproblems: (1) identifying the assignments of the trajectory of states TS that map to assignments of the trajectory of costs TC that satisfy the property; (2) computing the conditional probability of these assignments of TS with respect to the assignment of the trajectory of observations to. As we discussed in the section on general property checking, the first problem strictly depends on the nature of the property we are evaluating. The second problem, instead, can be efficiently tackled in its general form by combining different inference methods for HMMs, as shown in the following sections.

Traditional HMM inference

The most common algorithms for exact inference in HMMs are: the forward algorithm, the forward-backward algorithm and the Viterbi algorithm [3]. The first two answer queries about marginal probabilities while the third enables maximum *a posteriori* (MAP) inference [4]. More specifically, the forward algorithm can be used to compute the probability distribution of the current (or the upcoming) hidden variable, given a sequence of observations. This operation is often referred to as filtering (or prediction):

$$\begin{gathered} P(S_T \mid o_1, \ldots, o_T) \\ P(S_{T+1} \mid o_1, \ldots, o_T) \end{gathered} \tag{8}$$

The forward-backward algorithm allows to refine the estimate (smoothing) of the probability distribution of a hidden variable using subsequently collected information, that is, computing:

$$P(S_N \mid o_1, \ldots, o_T) \tag{9}$$

when $1 \leq N \leq T$. Finally, the Viterbi algorithm allows to discover the most likely sequence of assignments of the hidden variable for a given sequence of observations:

$$\underset{s_0,\ldots,s_T}{\operatorname{argmax}} P(s_0, \ldots, s_T \mid o_1, \ldots, o_T) \tag{10}$$

All these algorithms have time-complexity that is linear with the length of the sequence of observations they take as input: $O(T)$ [3]. However, none of these algorithms directly provides an answer to the family of queries that we are interest in for the scope of this work. That is, the *a posteriori* probabilities of arbitrary sequences of assignments of the hidden variable for a given sequence of observations, such as:

$$P(s_0, \ldots, s_T \mid o_1, \ldots, o_T) = P(ts|to) \tag{11}$$

(Note that the output of the Viterbi algorithm is only one, specific sequence of assignments and not a probability value.)

Efficient inference

The easiest, but highly inefficient, way to find the probability value of $P(ts|to)$ consists of computing the complete joint probability distribution of the c-HMM over the time horizon T. Because HMMs are Bayesian networks, their JPD is equal to the chain product of all the conditional probability distributions (CPDs) in their nodes. Then, one can condition by the evidence of to, and finally re-normalize the entire distribution so that it sums up to 1. This is clearly an unsustainably expensive approach because computing the JPD requires time and space complexity of $O(\Omega(S) \cdot \Omega(O))^T$.

Instead, we propose a much more efficient algorithm to compute the conditional probability of a finite state trajectory assignment $s_0, \ldots, s_T$ with respect to the observation trajectory assignment $o_0, \ldots, o_T$. To do so, we start by re-writing the probability value of interest using the Bayes' theorem:

$$P(s_0, \ldots, s_T \mid o_1, \ldots, o_T) = [P(o_0, \ldots, o_T \mid s_0, \ldots, s_T) \cdot P(s_0, \ldots, s_T)] \cdot P(o_0, \ldots, o_T)^{-1} = \Upsilon_0 \cdot \Upsilon_1 \cdot \Upsilon_2 \tag{12}$$

Eq 12 shows how to decompose the problem into the computation of three factors that we call Υ_0, Υ_1, and Υ_2 and can be tackled separately. Given an assignment of the

state variables, the conditional probability of a sequence of observations Υ_0 can be computed as the product of the appropriate entries of the sensor model.

$$\Upsilon_0 = P(o_0, \dots, o_T \mid s_0, \dots, s_T) = \prod_{i=1}^{T} P(O_t = o_i \mid S_t = s_i) \tag{13}$$

The probability of an assignment of the trajectory of states TS (notwithstanding the values taken by the observations variables) Υ_1, only depends on the transition model $P(S_{t+1} \mid S_t)$ and the ground belief $P(S_{-1})$: first, we need to compute the probability of S_0 taking the value of s_0 as $P(S_0 = s_0) = \sum_{\forall s \in \Omega(S)} P(S_{t+1} = s_0 \mid S_t = s) P(S_{-1} = s)$; then, the probability of the entire trajectory can be computed multiplying the appropriate entries of the transition model.

$$\Upsilon_1 = P(s_0, \dots, s_T) = P(S_0 = s_0) \cdot \prod_{i=1}^{T} P(S_{t+1} = s_i \mid S_t = s_{i-1}) \tag{14}$$

The computation of this latter factor, Υ_2, is the trickiest: it can be performed efficiently using a dynamic programming technique derived from the forward algorithm as explained in [5]. This technique iteratively computes the quantity $P(o_0, \dots, o_T)$—from now on re-written as $F(T, s_T)$—using the transition and sensor models. The initialization step of the algorithm is:

$$\forall x \in \Omega(S) \quad F(1, x) = P(S_0 = x) \cdot P(O_0 = o_0 \mid S_0 = x) \tag{15}$$

The distribution of $P(S_0)$ can be computed just like we did for Eq 14. The iteration step of the algorithm is:

$$\forall x \in \Omega(S) \quad F(t+1, x) = \sum_{y \in \Omega(S)} F(t, y) \cdot P(S_{t+1} = x \mid S_t = y) \cdot P(O_t = o_1 \mid S_t = x) \tag{16}$$

Finally, having iterated the algorithm until T, the inverse of Υ_2 is computed as the sum over all possible values of S_T:

$$\frac{1}{\Upsilon_2} = P(o_0, \dots, o_T) = \sum_{\forall x \in \Omega(S)} F(T, x) \tag{17}$$

With the values of the three factors Υ_0, Υ_1, and Υ_2, we can finally compute the

conditional probability of the assignment of a trajectory of states, given the assignment of a trajectory of observations as:

$$P(s_0, \ldots, s_T \mid o_0, \ldots, o_T) = \Upsilon_0 \cdot \Upsilon_1 \cdot \Upsilon_2 \tag{18}$$

The detailed analysis of the time- and space-complexity of the computation of all three factors is given in the main body of this article: the most relevant result is the fact that the overall time-complexity is linear w.r.t. the time horizon T.

Source code, scripts, and datasets

All the Octave scripts used to validate the proposed algorithm and perform the numerical simulations, a README file, as well as the LaTeXsource of the pseudo-code of the main inference algorithm are available as an open GIT repository at:
`https://github.com/JacopoPan/probabilistic-resilience`

References

1. Schwind N, Okimoto T, Inoue K, Chan H, Ribeiro T, Minami K, et al. Systems Resilience: A Challenge Problem for Dynamic Constraint-based Agent Systems. In: Proceedings of the 2013 International Conference on Autonomous Agents and Multi-agent Systems. AAMAS '13; 2013. p. 785–788. Available from: http://dl.acm.org/citation.cfm?id=2484920.2485043.

2. Ross SM. Introduction to Probability Models, Ninth Edition. Orlando, FL, USA: Academic Press, Inc.; 2006.

3. Russell SJ, Norvig P. Artificial Intelligence: A Modern Approach. 2nd ed. Pearson Education; 2003.

4. Koller D, Friedman N. Probabilistic Graphical Models: Principles and Techniques - Adaptive Computation and Machine Learning. The MIT Press; 2009.

5. Durand D, Hoberman R. Computational Genomics and Molecular Biology, HMM Lecture Notes. Carnegie Mellon University; 2011.
