# Supplementary figures and images for: Assessing the resilience of stochastic dynamic systems under partial observability
Source: PLoS One. 2018 Aug 23;13(8):e0202337. doi: 10.1371/journal.pone.0202337 (PMC6107160; doi:10.1371/journal.pone.0202337)

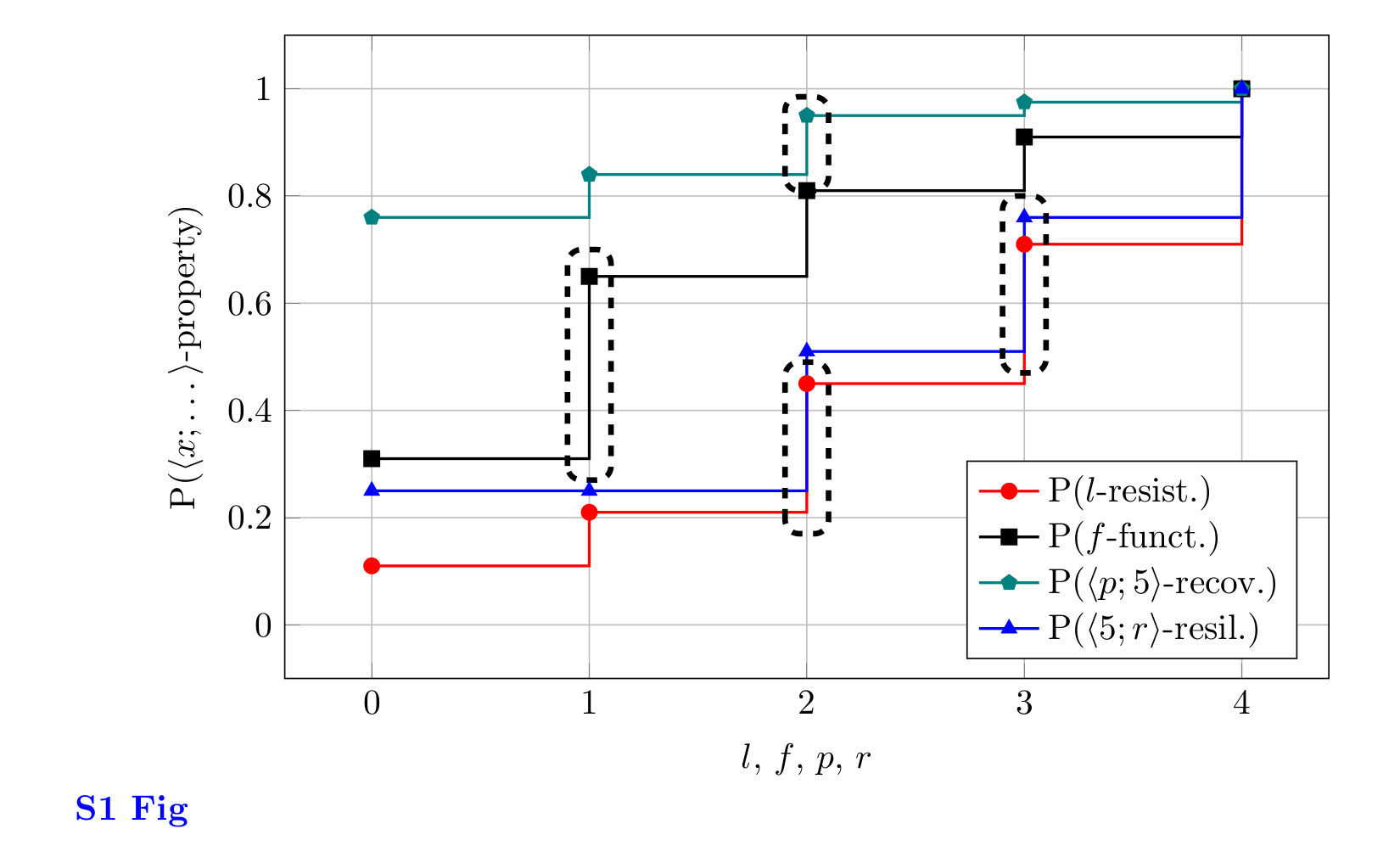

Supplement: S1 Fig — Probability distribution of the parametric resilient properties in a template scenario where ∀s, c(s) ∈ [0, …, 4]. The discontinuities reveal the potentially critical thresholds for different properties. (TIF) [file pone.0202337.s002.tif]
